# Lamarckian evolution explains human brain evolution and psychiatric disorders

**DOI:** 10.3389/fnins.2013.00224

**Published:** 2013-11-26

**Authors:** Guy Barry

**Affiliations:** Neuroscience Division, Garvan Institute of Medical ResearchDarlinghurst, NSW, Australia

**Keywords:** Lamarckian inheritance, pangenesis, psychiatric disease, vesicle, small non-coding RNA, *de novo* mutations

The current model of evolution, dominated by the Darwinian theory, is becoming increasingly improbable as a complete explanation of the forces driving human brain evolution. An underappreciated concept exists, proposed more than two centuries ago by Jean-Baptiste Lamarck, whereby somatic cells pass experience-dependent, and ultimately adaptive, information to subsequent generations. However, since no mechanism could be demonstrated, this theory was gradually displaced in favor of the more intellectually lenient modern evolutionary theory where powerful selection pressures result in the inheritance of beneficial genetic variations, randomly generated in the germline, that bestow greater fitness to subsequent generations. Recent evidence from studies, including psychiatric disease, suggests a mechanism whereby organisms constantly adapt to the environment and that this crucial information is passed to their offspring, rather than relying on a germline-independent and indiscriminate mechanism that takes no cues from the outside milieu. I propose that a combination of Darwin's hypothesis on pangenesis, coupled with Lamarckian somatic cell-derived epigenetic modifications and *de novo* RNA and DNA mutations, is the dominant mechanism of current cognitive evolution.

## Evolution of higher-order cognition

The human brain has remarkably tripled in size over the past five million years since the split from our nearest relative, the chimpanzee (Bradbury, 2005). The increased size of the neocortex, especially the prefrontal cortex (Balsters et al., 2010), is thought to provide a substrate for some of the enhanced cognitive abilities observed in humans (Kolb et al., 2012). Although some neural protein-coding genes have undergone some positive selection during this time (Dorus et al., 2004; Vallender et al., 2008), it is the expansion in the dynamically regulated non-protein-coding regions of the genome that display the most striking parallels with human brain progression (Taft et al., 2007). We have hypothesized that the non-coding repertoire, including various repeat elements, and RNA editing, have been the driving force for the increased flexibility of the brain resulting in stimulus-dependent adaptation ultimately leading to evolved higher-order cognition and, upon dysregulation, associated psychiatric disease (Barry and Mattick, 2012). However, this does depend on a mechanism allowing the transgenerational transmission of adaptive neuronal changes from the somatic cells of the brain. It is likely that both coding (proteins) and non-coding (regulatory effectors) regions of the genome undergo adaptive change to external stimuli and that these changes will be reflected in the transcriptomic output of the specific cell. But would experience-dependent *de novo* changes be beneficial to the evolution of an organism and, if so, how are they transmitted to the germline?

## *De novo* mutations underlie psychiatric disorders

Psychiatric disorders such as autism, schizophrenia, and intellectual disability offer valuable insight into current evolutionary mechanisms. The recent advent of next generation sequencing, especially where parents and affected and non-affected siblings were sequenced to detect *de novo* mutations, has allowed an unprecedented view of whole genome transcriptomics in human brain evolution (Johnson et al., 2009). Strikingly, although the prevalence of individual *de novo* mutations in autism is extremely low, the mutations seem to cluster to specific genetic pathways (Neale et al., 2012; O'Roak et al., 2012a,b). Copy number variations arising from *de novo* mutations also play a significant role in sporadic cases of autism (Itsara et al., 2010; Sanders et al., 2011; Devlin and Scherer, 2012) and schizophrenia (Rees et al., 2012). Furthermore, intellectual disability is strongly associated with *de novo* mutations (Girirajan et al., 2011; de Ligt et al., 2012; Rauch et al., 2012; Veltman and Brunner, 2012) reinforcing these mechanisms as causative in cognitive disorders. As more studies are performed on patients with psychiatric disorders it is becoming apparent that there is a significant overlap in gene pathways affected by *de novo* mutations (Gilman et al., 2012). It must be noted that this process is in all likelihood not entirely deleterious because if mutations arose that were neutral or advantageous they would not be studied to nearly the same degree as those associated with negative effects. Future studies investigating higher cognitive abilities in “normal” subjects would be of great interest to the field. I believe that this reveals the current evolutionary phase of stimulus-dependent adaptation to enhance cognition. Overall, these data provide strong evidence that brain evolution is, at least partly, not random, but instead driven by targeted *de novo* genetic mutations and inherently assumes a method of somatic transmission due to high heritability rates (Yu et al., 2013).

The increased risk of psychiatric diseases, such as autism and schizophrenia, with increasing paternal age (Kong et al., 2012) suggests that accumulating *de novo* mutations in the brain are heritable and predominantly arise from sperm. The age-related decrease in sperm quality does not seem to be an adequate explanation for this as the mutations cluster non-randomly in specific genetic pathways. Maternally transmitted epigenetic inheritance undoubtedly occurs during maternal exposure to adverse conditions, such as stress and obesity, during pregnancy (Dunn et al., 2011; Matthews and Phillips, 2012) and in some cases of autism (Cook et al., 1997; Noor et al., 2010). However, due to the limited number of eggs contained in ovaries, and therefore, the limited scope for non-stress related experimentation, it would seem reasonable that the almost limitless number of sperm would be better suited to testing the consequences of directed *de novo* mutations. Moreover, epigenetic effects that transfer from the sperm is more acceptable since the possibility that the eggs themselves are exposed to the stress are excluded.

## Transgenerational epigenetic inheritance

The field of epigenetics has recently revealed evidence of experience-dependent transgenerational inheritance. The modern evolutionary theory proposes that evolution occurs through a combination of Mendelian genetics and Darwin's theory of natural selection (Kutschera and Niklas, 2004). However, examples contradicting this theory have emerged such as the contribution of modifiable epigenetic processes that do not disturb DNA sequence (Danchin et al., 2011). Epigenetic mechanisms involved in inheritance include DNA methylation, histone modifications, and small non-coding RNA (sncRNA)-driven mechanisms, e.g., small interfering RNA (siRNA), piwi-interacting RNA (piRNA), and microRNA (miRNA). Epigenetic alterations contribute to transgenerational inheritance in plants, such as during adaptive plasticity in response to pathogenic invasion (Holeski et al., 2012). Likewise, analogous epigenetic mechanisms transmit potentially somatic experience-dependent information across generations in *C. elegans* (Rechavi et al., 2011; Ashe et al., 2012). Although the demonstration of specific sncRNA-dependent mechanisms involved in transgenerational inheritance are scarce in mammals it is clear that transgenerational inheritance does indeed occur in mammals (Rassoulzadegan et al., 2006; Daxinger and Whitelaw, 2012; Saavedra-Rodriguez and Feig, 2013; Vassoler et al., 2013), including in the brain (Bohacek et al., 2013). Furthermore, unlike plants, metazoans possess an extra class of small RNAs, piRNAs that, although initially thought to be germ cell-specific, are present in the brain (Rajasethupathy et al., 2012) and have been demonstrated to be involved in transgenerational inheritance (Ashe et al., 2012; Grentzinger et al., 2012). The transfer of somatic information from organs, like the brain, to the testis would require that separate organs are capable of the same processes (to ensure equivalent transcriptional regulation) as is the case for sncRNAs as these mechanisms are present in the germline (Saxe and Lin, 2011; Ashe et al., 2012; Buckley et al., 2012) and in the brain (Qureshi and Mehler, 2012).

Although not yet demonstrated, sncRNAs would additionally require the ability to induce DNA changes, either directly or indirectly, to influence inheritance patterns of *de novo* mutations in psychiatric disease uncovered through exome sequencing (as discussed above). At least two mechanisms are possible; First, sncRNAs are able to induce a DNA change through regulating DNA repair (Francia et al., 2012; Chowdhury et al., 2013) and, second, determining site-selectivity for generic DNA editing enzymes, such as the APOBEC family (Smith et al., 2012), some members of which are recently implicated in psychiatric disease (Dong et al., 2012). It is also been suggested that these two mechanisms function together in DNA recoding to enhance cognitive evolution (Mattick and Mehler, 2008).

## Possible mechanism of transmission of lamarckian inheritance

The Lamarckian theory of inheritance has historically been rejected due to a lack of a confirmed mechanism. However, I believe that long-range RNA-mediated information transmission is best suited to fill this role. In plants, regulatory information, conveyed through stimulus-dependent transcription of small non-coding RNAs (Sunkar and Zhu, 2004; Mallory and Vaucheret, 2006) and their long-distance transport throughout the plant (Yoo et al., 2004; Chuck and O'Connor, 2010), has been harnessed for the transmission of adaptive somatic cell responses. The utilization of RNA as a method of communication between cells is present in all organisms, including humans, where regulatory information is passed locally and systemically within vesicles (Thery et al., 2002; Fevrier and Raposo, 2004; Ratajczak et al., 2006; Dinger et al., 2008; Muralidharan-Chari et al., 2010). Extracellular vesicles have been shown to pass through the blood—brain barrier, and are similarly likely to pass through the blood—testis barrier, and may be of significant clinical relevance for disease biomarkers and therapeutic delivery due to this unique endogenous ability (van der Pol et al., 2012). Indeed, “biomarkers” may exist in the bloodstream due to the active process of long-range communication between somatic cells, and somatic cells and the germline. Recent reports suggest blood biomarkers can be used to detect psychiatric disorders such as schizophrenia (Schwarz et al., 2012), autism (Momeni et al., 2012), and may predict suicidality (Le-Niculescu et al., 2013), possibly reflecting a snapshot of real-time cellular transcription that would incorporate disease-associated sporadic *de novo* mutations.

Charles Darwin proposed that there existed particles of inheritance, called “gemmules” or “pangenes,” that carried information of inheritance to the sexual elements and that these particles may be modifiable by the environment (Darwin, 1868). I propose that these particles are in fact vesicles, released by many tissues into the bloodstream (van der Pol et al., 2012) and that they provide an attractive means of transmission for RNA-mediated somatic cell-derived regulatory information to the germline. Vesicles contain mostly sncRNA (Lasser et al., 2012), especially miRNA (Guduric-Fuchs et al., 2012) that may be differentially expressed in psychiatric disease (Banigan et al., 2013). Vesicles have also been demonstrated to relay RNA-coded information between neighboring neural cells (Fruhbeis et al., 2012) but definitive evidence for long-range RNA communication in mammalian systems has not yet been produced.

Recent ENCODE data reveal that testes show the highest quantity of transcription in the body (Howald et al., 2012) and, I postulate, that this may reflect the overall transcription from all other body regions. Brain tissue shows the second-highest rate of transcription and all other tissues are by a large extent less. I further suggest that this high level of transcription in the brain reflects the current state of evolution, where the human brain is undergoing extraordinary accelerated environment- and stimulus-dependent adaptive evolution (as discussed above) and that this information is transferred to the testes for inheritance. Other tissues are concomitantly undergoing adaptive responses, albeit at normally lower levels, and that this information is also routed to the testes resulting in a higher overall rate of transcription in the testes compared with brain.

## Concluding remarks and future directions

I propose that the human brain is undergoing an evolutionary explosion, with non-coding RNA-mediated mechanisms (allowing extraordinary adaptive plasticity) and somatic cell-derived, Lamarckian-type inheritance, driving this process. The prevailing hypothesis that brain evolution, or in fact any adaptive form of transgenerational inheritance, is independently controlled solely by a Darwinian method of random mutations and epigenetic modifications in the germline is highly unlikely as specific responses to many types of stressors, from plants through to humans, are robustly transmitted to subsequent generations (Rassoulzadegan et al., 2006; Rechavi et al., 2011; Ashe et al., 2012; Daxinger and Whitelaw, 2012; Holeski et al., 2012; Bohacek et al., 2013; Saavedra-Rodriguez and Feig, 2013; Vassoler et al., 2013).

There is little doubt that in the near future a mechanism of Lamarckian inheritance, possibly mediated by small regulatory RNA-containing vesicles, will be uncovered. However, before the wider scientific community accepts this hypothesis, it will be crucial to show vesicle transport, together with RNA cargo, between brain and testis, and specific stimulus-driven transcription in somatic cells reflected in the germline and subsequent inherited DNA and/or epigenetic modifications.

Darwin did not discard Lamarck's ideas on evolutionary mechanisms and was open to the idea of pangenesis where “gemmules” or “pangenes” would transmit somatic information to the germline. I believe that these exist as vesicles in the blood and that Lamarck's hypothesis on somatic inheritance coupled with Darwin's mechanism of pangenesis will be established as a more complete model of forces driving human brain evolution.
